# The Effect of Sibunit Carbon Surface Modification with Diazonium Tosylate Salts of Pd and Pd-Au Catalysts on Furfural Hydrogenation

**DOI:** 10.3390/ma15134695

**Published:** 2022-07-04

**Authors:** Dmitrii German, Ekaterina Kolobova, Ekaterina Pakrieva, Sónia A. C. Carabineiro, Elizaveta Sviridova, Sergey Perevezentsev, Shahram Alijani, Alberto Villa, Laura Prati, Pavel Postnikov, Nina Bogdanchikova, Alexey Pestryakov

**Affiliations:** 1 Research School of Chemistry and Applied Biomedical Sciences, National Research Tomsk Polytechnic University, Lenin Av. 30, 634050 Tomsk, Russia; germandmitry93@gmail.com (D.G.); ekaterina_kolobova@mail.ru (E.K.); epakrieva@mail.ru (E.P.); evs31@tpu.ru (E.S.); postnikov@tpu.ru (P.P.); 2 Centro de Química Estrutural, Institute of Molecular Sciences, Departamento de Engenharia Química, Instituto Superior Técnico, Universidade de Lisboa, Av. Rovisco Pais, 1049-001 Lisboa, Portugal; sonia.carabineiro@fct.unl.pt; 3 LAQV-REQUIMTE, Department of Chemistry, NOVA School of Science and Technology, Universidade NOVA de Lisboa, 2829-516 Caparica, Portugal; 4 Institute of Petroleum Chemistry, Russian Academy of Science, Akademichesky Av. 4, 634021 Tomsk, Russia; sap311@yandex.ru; 5 Dipartimento di Chimica, Università degli Studi di Milano, via Camillo Golgi 19, 20133 Milano, Italy; sli@dinex.fi (S.A.); alberto.villa@unimi.it (A.V.); laura.prati@unimi.it (L.P.); 6 Centro de Nanociencias y Nanotecnología, Universidad Nacional Autónoma de México, Ensenada 22800, Mexico; nina@cnyn.unam.mx; 7 Laboratory of Catalytic and Biomedical Technologies, Sevastopol State University, 299053 Sevastopol, Russia

**Keywords:** gold, palladium, Sibunit carbon, bimetallic catalysts, surface modification, tosylate salts, hydrogenation, furfural, furfuryl alcohol

## Abstract

Herein, we investigated the effect of the support modification (Sibunit carbon) with diazonium salts of Pd and Pd-Au catalysts on furfural hydrogenation under 5 bars of H_2_ and 50 °C. To this end, the surface of Sibunit (Cp) was modified with butyl (Cp-Butyl), carboxyl (Cp-COOH) and amino groups (Cp-NH_2_) using corresponding diazonium salts. The catalysts were synthesized by the sol immobilization method. The catalysts as well as the corresponding supports were characterized by Fourier transform infrared spectroscopy, N_2_ adsorption-desorption, inductively coupled plasma atomic emission spectroscopy, high resolution transmission electron microscopy, energy dispersive spectroscopy, X-ray diffraction, Hammet indicator method and X-ray photoelectron spectroscopy. The analysis of the results allowed us to determine the crucial influence of surface chemistry on the catalytic behavior of the studied catalysts, especially regarding selectivity. At the same time, the structural, textural, electronic and acid–base properties of the catalysts were practically unaffected. Thus, it can be assumed that the modification of Sibunit with various functional groups leads to changes in the hydrophobic/hydrophilic and/or electrostatic properties of the surface, which influenced the selectivity of the process.

## 1. Introduction

Carbon-based materials have captured significant attention of the scientific community for decades due to their interesting properties, such as superb chemical and mechanical dependability, large surface areas and pore volumes, lightweight nature, variable structural and morphological combinations, mass-scale availability, excellent recyclability, and low production cost. This is reflected in the targeted synthesis of allotropic forms of carbon (carbines, fullerenes, nanotubes, circulenes, etc.), as well as in the creation of a wide range of porous materials in a series of mixed (transitional) forms of carbon [1,2,3].

Carbon-based materials can be successfully used as structural modifiers of construction materials, electronic elements, hydrogen accumulators, and additives to lubricants, varnishes and paints, gas distribution layers of fuel cells, and high-performance adsorbents. The use of carbon nanostructures in fine chemical synthesis, biology, and medicine is also widely discussed [4,5,6].

Notably, carbon materials are suitable catalyst supports for metal nanoparticles, (NPs) owing to advantages such as developed porous space to transfer reactants and reaction products, chemical inertness (especially in the presence of strong acids and bases), mechanical stability, structural diversity, and controlled chemical surface properties [7,8,9]. Among the variety of carbon supports for heterogeneous catalysts, the Sibunit material attracts particular attention because it has a unique combination of properties of graphite (chemical stability) and activated carbon (high specific surface and sorption capacity). Palladium heterogeneous catalysts deposited on Sibunit were applied in industrial processes of hydropurification of terephthalic acid, hydrogenation of m-nitrobenzene trifluoride and o-nitrophenol, and in rosin disproportionation [10].

At the same time, carbon materials free from surface functional groups are known to be hydrophobic or non-polar, which can lead to weak stabilization of metal NPs on the carbon support. Therefore, these factors determine the sorption interaction of the active component with the support. Besides metal dispersion enhancement, the functionalities on the carbon surface can act as anchoring sites in the synthesis of carbon-based composite materials; and carbon surface modification can change the electronic surface state and the contribution of the active state of the deposited metal, modify the acid–base properties, etc., [11,12].

Thus, chemical functionalization can be used to tailor the surface physicochemical properties by applying appropriate thermal or chemical treatments or by anchoring the desired functional groups.

Among the functionalization approaches, diazonium chemistry is becoming increasingly attractive as this promising method, which combines the ease of preparation of diazonium salts, rapid reduction, and strong aryl–substrate surface atoms covalent bonding, allows grafting different organic moieties onto various solid supports, such as carbon material [13,14,15]. Among all types of diazonium salts, arenediazonium tosylates can be considered as the most convenient reagents for the surface modification, due to their stability, inexplosive properties and good solubility in water [16]. Moreover, the arenediazonium tosylates have been widely applied for the surface modification of metal and carbon surfaces [17,18].

Surface functionalization by incorporation of other elements (heteroatoms) or functional groups (which are formed from these heteroatoms) is believed to be essential for the enhancement of the catalytic activity of carbon-containing materials. For instance, an increase in the basic properties via introducing nitrogen-containing groups of the support might be beneficial in liquid phase oxidation or hydrogenation. The previous paper demonstrated that the presence of N on the carbon surface can influence the oxidation state of active sites (Pd^2+^ and Au^+^) and, consequently, led to higher desired acid production, derived from 5-hydroxymetylfurfurol oxidation [19]. Amadou et al. investigated Pd on nitrogen-doped carbon nanotubes for the selective hydrogenation of cinnamaldehyde into hydrocinnamaldehyde [20]. The high TOF and high selectivity towards the C=C bond hydrogenation were attributed to possible electronic or morphological modifications that occurred after nitrogen atom incorporation. Acidic groups, such as carboxyl, quinone and lactone, provide interaction between the carbon surface and positively charged cations of the precursor metal. In addition, they reduce the hydrophobicity of carbon, thus making the surface more accessible for aqueous solutions of precursors. Liang et al. found that the acidic functional groups (carboxyl groups) of activated carbon, used as the support for Pd catalysts, enhanced the content of Pd^2+^, benefited the dispersion of Pd, and eventually improved the H_2_O_2_ selectivity from H_2_ and O_2_ [21]. Phenol, carbonyl and ether groups are slightly acidic or neutral. For example, Bianchi et al. found that the activity in the liquid-phase oxidation of ethylene glycol on Au/C with similarly sized Au particles increases by increasing the amount phenol-type groups, indicating that a specific metal–support interaction does exist [22].

In this study, the hydrogenation of furfural was used as a model reaction due to the wide range of possible products, which allows us to clearly demonstrate the possibility of varying the selectivity by changing the surface chemistry. Moreover, by hydrogenation of furfural, organic compounds, such as furfuryl alcohol, tetrahydrofurfuryl alcohol, 2-methylfuran and 2-methyltetrahydrofuran, can be obtained, which are alternative sources of organic substances [23]. Furfural hydrogenation products are in demand as environmentally friendly solvents, in the polymer and coating industry, fuel additives, pharmaceuticals, etc [24,25,26]. Table 1 shows results for hydrogenation of furfural over various heteroheneous catalysts.

Thus, the preparation of highly dispersed, efficient and selective metal catalysts relies heavily on the proper control of the surface chemistry of the carbon support, which is a great scientific and practical interest in the last few years for the development of advanced catalytic systems for different conversions. This study aims to show how the catalytic behavior of mono- and bimetallic catalysts for furfural hydrogenation can be influenced by modifying the surface of the carbon material with diazonium tosylate salts.

## 2. Materials and Methods

The reagents (furfural, sodium tetrachloropalladate (II) (Na_2_PdCl_4_), sodium tetrachloroaurate (III) dehydrate (NaAuCl_4_·H_2_O), polyvinyl alcohol (PVA), sulfuric acid (H_2_SO_4_), sodium borohydride (NaBH_4_)) from Merck (Darmstadt, Germany) were used. The carbon material Sibunit was purchased from the Center of New Chemical Technologies of Boreskov Institute of Catalysis (Omsk, Russia).

**Figure 1:**
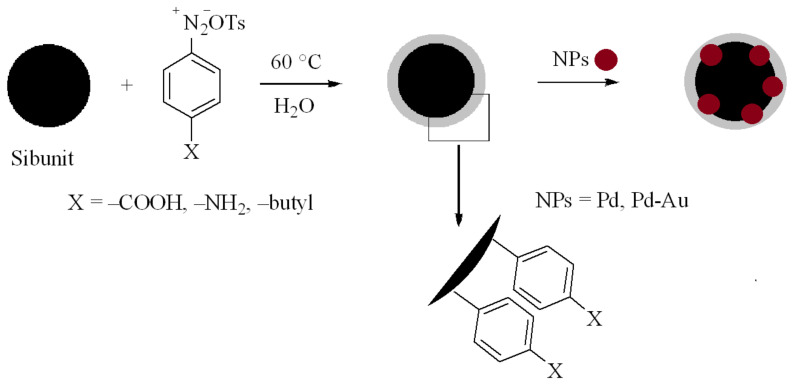
Scheme of support modification by diazonium salts and immobilization of Pd and Au nanoparticles.

The diazonium tosylate (4-carboxybenzenediazonium tosylate, 4-butylbenzenediazonium tosylate or 4-aminobenzenediazonium tosylate) was prepared according to the published procedure [33,34]. The covalent modification was carried out by the immersion of Sibunit in the solution of diazonium salts as follows: the carbon material Sibunit (denoted hereinafter as Cp) (1 g) was dispersed in 5 mL of water/methanol (4/1) solution and 1 mmol of the corresponding diazonium tosylate (4-carboxybenzenediazonium tosylate, 4-butylbenzenediazonium tosylate or 4-aminobenzenediazonium tosylate) was added to solution. The mixture was sonicated for 20 min and then stirred for 60 min at 60 °C. Afterwards, the modified powders were sequentially rinsed under sonication with deionized water, ethanol, and methanol for 10 min and dried in a desiccator for 3 h (Figure 1).

The monometallic catalysts (Pd/Cp, Pd/Cp-COOH, Pd/Cp-butyl and Pd/Cp-NH_2_) were synthesized by the sol immobilization method [35]. A total of 1 mL Na_2_PdCl_4_ solution (10 mg Pd/mL H_2_O) and 0.5 mL PVA solution (1 wt. %) were added to 100 mL miliQ H_2_O under vigorous stirring. After a few minutes, a solution of NaBH_4_ (Pd/NaBH_4_ = 1/8 mol/mol) was added to form a brown palladium sol. Then, the support was added into the sol solution (assuming that the catalyst would contain 1 wt. % palladium) and a few drops of H_2_SO_4_ (to immobilize palladium nanoparticles on the support). The synthesis was carried out for 1 h. The resulting catalyst washed and dried at 80 °C for 2 h under air.

The bimetallic catalysts (Pd-Au/Cp, Pd-Au/Cp-COOH, Pd-Au/Cp-butyl and Pd-Au/Cp-NH_2_) were prepared by the same method as the monometallics. A total of 0.684 mL Na_2_PdCl_4_ (10 mg Pd/mL H_2_O), 0.316 mL NaAuCl_4_·H_2_O (10 mg Au/mL H_2_O) and 0.5 mL PVA solution (1 wt. %) were added to 100 mL miliQ H_2_O under vigorous stirring. After a few minutes, a solution of NaBH_4_ (metal/NaBH_4_ = 1/8 mol/mol) was added and a brownish-red palladium-gold sol was formed. Then, the support was added in the sol solution (assuming that the catalyst would contain 1 wt.% of metals with a ratio of Pd:Au = 4:1 mol/mol) and several drops of H_2_SO_4_ (for immobilization of palladium and gold nanoparticles on the support). The synthesis was carried out for 1 h. The resulting catalyst was washed and dried at 80 °C for 2 h under air.

Fourier transform infrared (FTIR) spectra were recorded using a ThermoScientific FTIR instrument (Nicolet 8700) to confirm the functionalization of the Sibunite surface. Palladium and gold content were measured by inductively coupled plasma atomic emission spectroscopy (ICP-AES) using iCAP 6300 Duo (Thermo Fisher Scientific, Waltham, MA, USA). XRD patterns were registered by Bruker D8 X-ray diffractometer (Bruker Corporation, Billerica, MA, USA) for the identification of the phase composition of catalysts. The textural properties were measured using an ASAP 2060 (Micromeritics Instrument Corporation, Norcross, GA, USA) apparatus. The acid–base properties of the supports and the corresponding catalysts were studied by the Hammett indicator method. The sizes and distribution of Pd or Pd-Au particles were estimated by high resolution transmission electron microscopy (HRTEM) using a JEOL JEM-2100F (JEOL Ltd., Tokyo, Japan). Surface composition and the chemical state of each element were determined by X-ray photoelectron spectroscopy (XPS), performed on a VG Scientific ESCALAB 200A (Thermo Fisher Scientific, Waltham, MA, USA). Detailed information is available in the Supplementary Materials.

**Figure 2:**
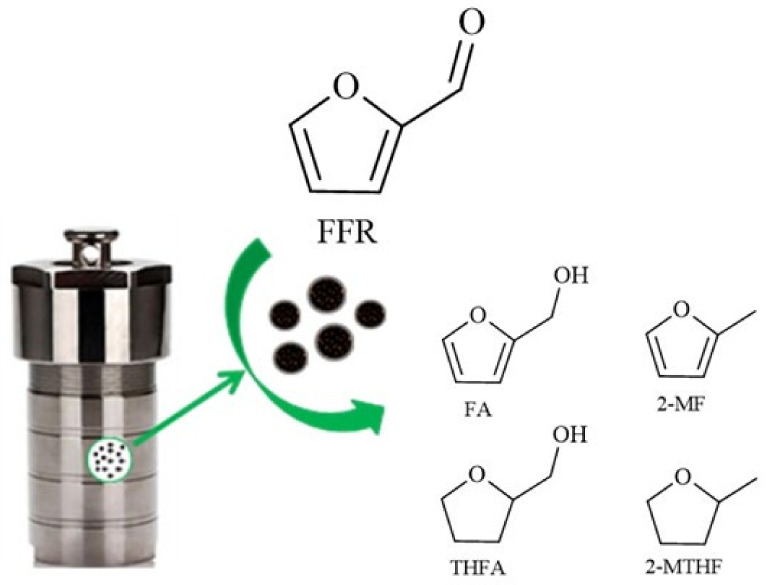
Schematic representation of the catalytic process.

The reactions of hydrogenation were carried out under 0.5 MPa pressure of H_2_, at 50 °C and stirring at 1200 rpm (Figure 2). The molar ratio of metal: furfural was 1:500 for monometallic and 1:580 for bimetallic systems. The catalytic reactions were performed in a 50 mL stainless steel batch reactor equipped with a heater and a stirrer. Firstly, 10 mL of a 0.3 M solution of furfural in isopropanol and catalyst were mixed into the reactor, and then the system was purged several times with nitrogen and hydrogen. To monitor the progress of the reaction, aliquots were taken at certain time intervals and analyzed using an Agilent 6890 gas chromatograph (Agilent, Santa Clara, CA, USA) equipped with a Zebron ZB-5 column 60 m × 0.32 mm × 1 μm.

## 3. Results

### 3.1. FTIR Results

**Figure 3:**
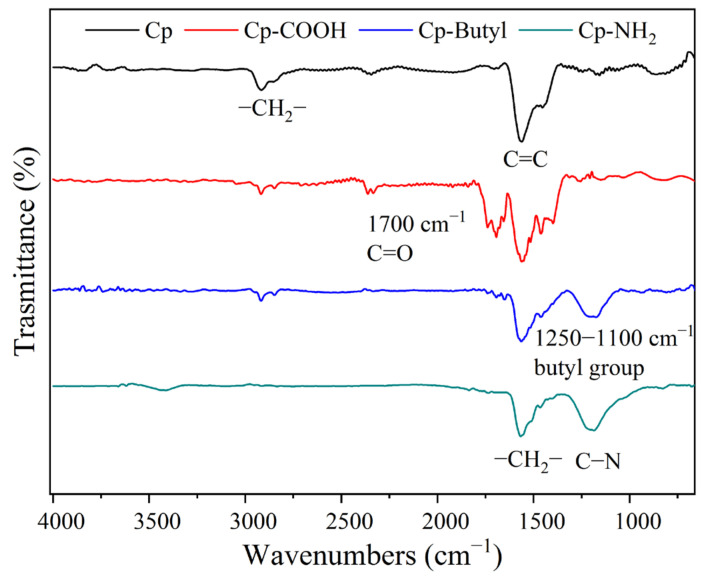
FTIR spectra of Cp, Cp−COOH, Cp−butyl and Cp−NH_2_.

A scheme dealing with the functionalization is shown in Figure 1. The surface modification by butylphenyl, aminophenyl and carboxyphenyl groups was proved by Fourier transform infrared (FTIR) spectroscopy. FTIR spectrum of Cp exhibits vibration bands associated with the graphitic carbon structure (C=C bond at 1600 cm^−1^, CH_3_ group at 1440–40 cm^−1^). After modification, novel vibration bands associated with the covalent attachment of amino, carboxyl or butyl groups appeared, related to the stretching vibrations of C=O (1700 cm^−1^), butyl aliphatic stretch (1100–1250 cm^−1^) and C–N stretch (1300–1100 cm^−1^) (Figure 3).

### 3.2. ICP-AES and XEDS Results

Palladium and gold contents (Table 2) were determined by inductively coupled plasma atomic emission spectroscopy (ICP-AES) and energy dispersive spectroscopy (XEDS). The presented data show that the Pd and Au contents are close to the nominal values for the entire series of catalysts.

### 3.3. XRD Results

**Figure 4:**
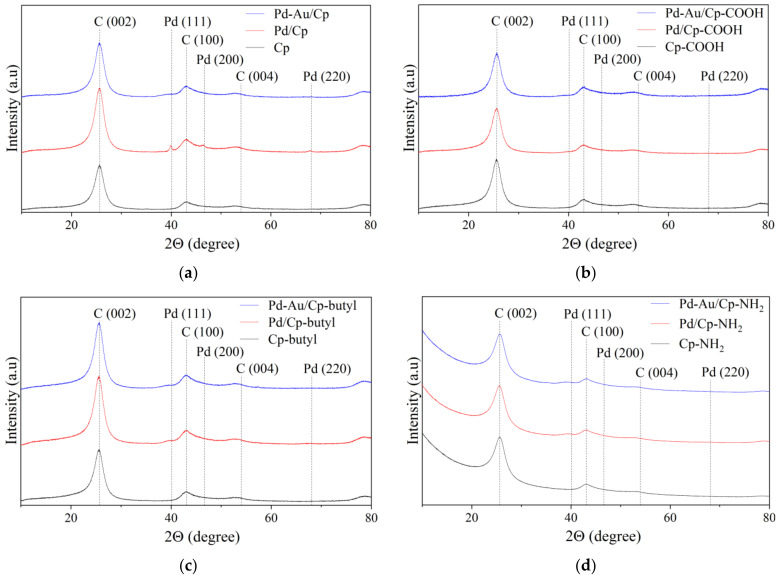
XRD patterns of catalysts and corresponding supports: (**a**) Cp, Pd/Cp and Pd-Au/Cp; (**b**) Cp-COOH, Pd/Cp-COOH and Pd-Au/Cp-COOH; (**c**) Cp-butyl, Pd/Cp-butyl and Pd-Au/Cp-butyl; (**d**) Cp-NH_2_, Pd/Cp-NH_2_ and Pd-Au/Cp-NH_2_.

**Figure 5:**
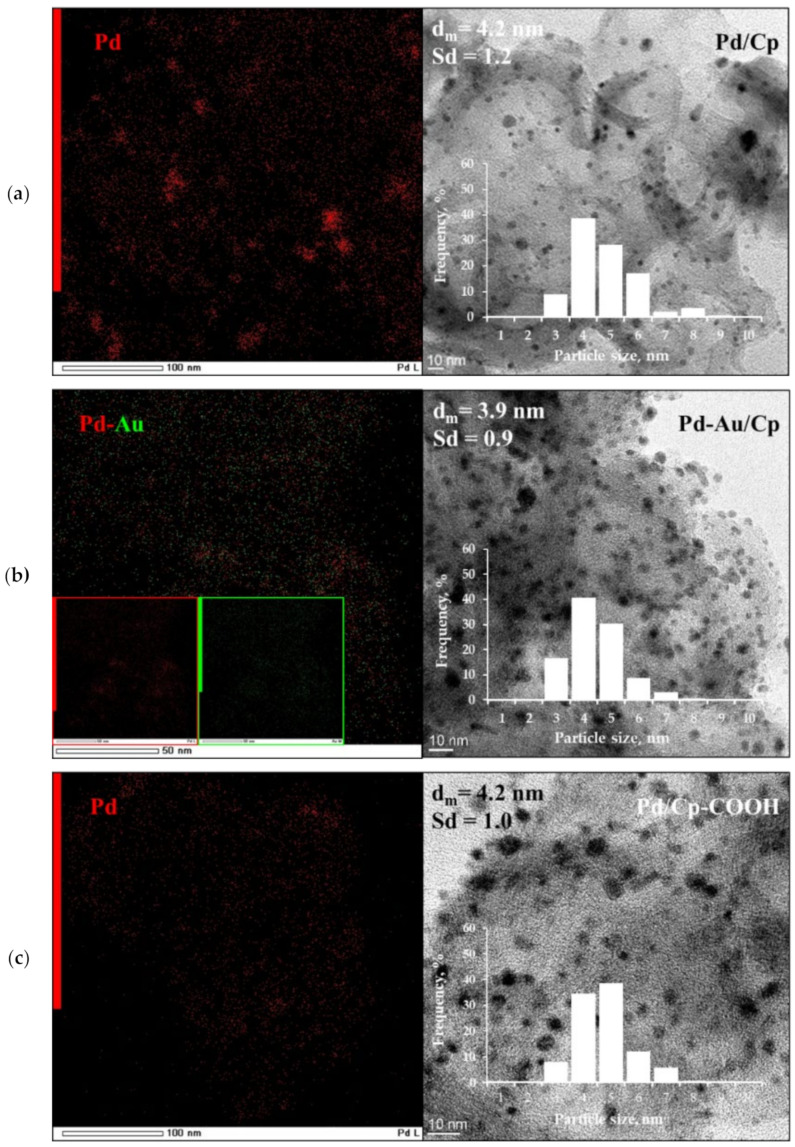

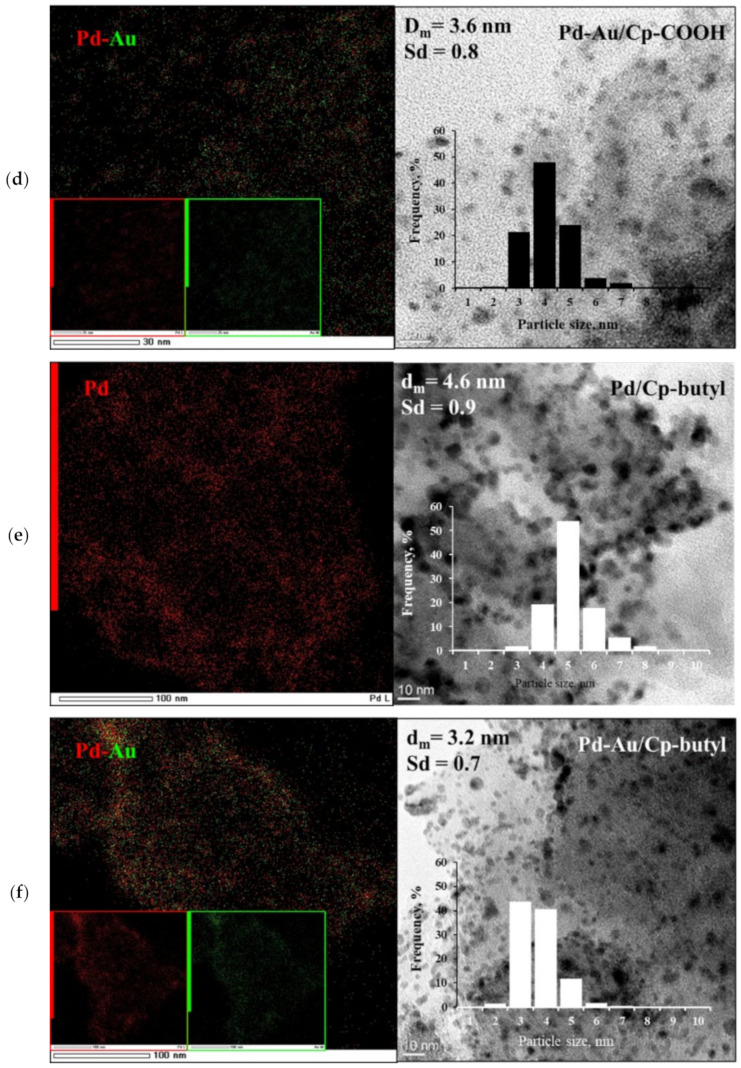

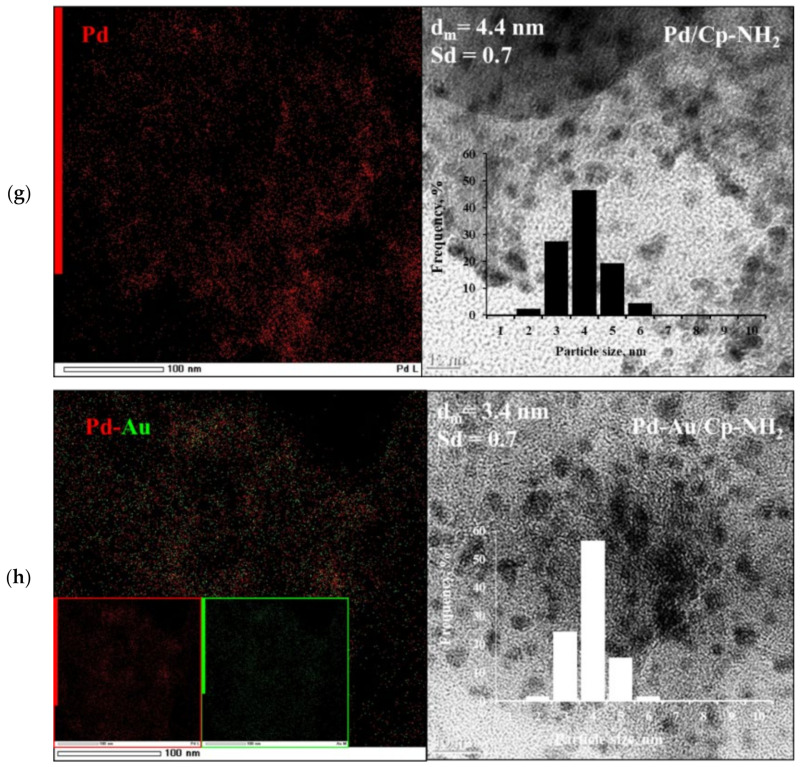
TEM images and EDX maps of the studied catalysts, as well as Pd and Pd-Au NPs distribution: (**a**) Pd/Cp; (**b**) Pd-Au/Cp; (**c**) Pd/Cp-COOH; (**d**) Pd-Au/Cp-COOH; (**e**) Pd/Cp-butyl; (**f**) Pd-Au/Cp-butyl; (**g**) Pd/Cp-NH_2_; (**h**) Pd-Au/Cp-NH_2_.

The phase composition of the catalysts and corresponding supports was studied using X-ray diffraction (Figure 4). The analysis of the spectra showed the absence of palladium and gold reflections for all samples, except Pd/Cp. For the latter, maxima were found in the XRD pattern at 2θ = 40.1°, 46.6°, and 68.1°, which are typical for palladium with a face-centered cubic lattice [36]. The appearance of the reflex for Pd/Cp is probably related to the non-uniform distribution of Pd NPs on the support surface resulting in their local accumulation, as evidenced by EDX maps (Figure 5). The absence of reflections in the X-ray diffraction patterns of the remaining samples is probably due to the small size of palladium and gold particles (below sensitivity of the XRD method) or their X-ray amorphous structure. The reflections of the support (2θ = 25.7°, 44.3°) correspond to multi-walled carbon nanotubes, which is consistent with the literature data [37,38].

### 3.4. Textural Properties

According to the data presented in Table 3, the specific surface area (S_BET_) is reduced by 26, 12 and 16%, respectively (Table 3, entries 1, 4, 7 and 10) as a result of the Sibunit modification by carboxyl, butyl and amino groups. Taking into account that the average cross section of a nitrogen molecule is 3.2 Å, the presence of functional groups at the entrance to the pores will prevent their filling with gas, thereby leading to a decrease in the surface available for adsorption [39,40,41]. This effect is more pronounced for pores lower than 2 nm. An important role in the manifestation of this effect is played by the nature of the functional groups. In the present study, it is clearly demonstrated by the example of a decrease in the specific surface of micro- and mesopores, as well as an increase in the average size of mesopores, indicating the blockage of small mesopores (Table 3). Separately, it should be noted that in the case of modification of Sibunit by carboxyl groups, all micropores become inaccessible to nitrogen molecules (Table 3, entry 4).

The deposition of palladium on the surface of unmodified and carboxyl-modified Sibunit resulted in an increase in S_BET_ by 10% and 8%, respectively, compared to the corresponding supports (Table 3, entries 1, 2, 4 and 5). An increase in the specific surface area after the deposition of palladium may indicate a small size of Pd nanoparticles (Pd NPs), a defective structure of NPs and their uniform distribution over the support surface, thereby providing new adsorption sites. In the case of a support modified by butyl groups, the application of palladium does not significantly change the value of the specific surface area compared to the corresponding support (Table 3, entries 7 and 8). When palladium is supported on Sibunit modified by amino groups, the specific surface area does not change (Table 3, entries 10 and 11).

Bimetallic catalysts are characterized by higher S_BET_ compared to the corresponding monometallic catalysts and supports (Table 3, entries 3, 6, 9 and 12). This may be a consequence of the formation of additional adsorption sites, due to the uniform distribution and small size of Pd-Au NPs. It is also necessary to consider possible changes in the surface of the supports under the action of the reagents used in the process of catalyst synthesis. For all investigated catalysts (Table 3, entries 2, 3, 5, 6, 8, 9, 11 and 12), the average sizes and volumes of micro- and mesopores vary from 1.8 to 2.0 nm and from 5.9 to 6.9 nm, from 0.001 to 0.003 cm^3^/g and from 0.45 to 0.60 cm^3^/g, respectively. In this case, the observed changes in the size and volume of the pores may be due to the impact of the reagents used for the synthesis of catalysts, as well as pore blocking by metal nanoparticles, which can serve as new adsorption sites.

### 3.5. Acid-Base Properties

The distribution and concentration of acid–base sites on the surface of supports and corresponding catalysts are presented in Table 4.

For Lewis basic sites (LBS), the highest content of LBS was detected on the surface of samples modified by amino groups, including the support and the corresponding mono- and bimetallic catalysts, followed by unmodified samples, materials modified by carboxyl groups, except the support, and samples containing butyl groups. Within the same group of samples. The concentration of LBS is practically unchanged.

For Brønsted acid sites (BAS), the highest content of BAS was detected on the surface of unmodified and amino-modified samples, except for Cp-NH_2_, followed by samples modified by carboxyl and butyl groups. The concentration of BAS for the unmodified samples varies slightly between the support and the corresponding catalysts. For samples modified by carboxyl and amino groups, the BAS concentration increases after palladium or bimetallic Pd-Au system deposition and decreases for samples containing a butyl group.

For Brønsted basic sites (BBS), the highest content of BBS was detected on the surface of unmodified samples. At the same time, the concentration of BBS for support and corresponding mono- and bimetallic catalysts is practically the same. The samples modified by carboxyl and butyl groups follow in terms of BBS content. The concentration of BBS on the surface of these samples increases in the following order: supports < monometallic catalysts < bimetallic catalysts. In the case of samples modified by amino groups, the highest BBS content is observed on the surface of the support; after palladium or bimetallic deposition, it decreases by more than 2 fold.

For Lewis acid sites (LAS), the highest LAS content was detected on the surface of unmodified and amino-modified samples. Moreover, the LAS concentration changes slightly after Sibunit modification by amino groups or deposition of palladium and bimetallic Pd-Au system. For samples containing a butyl functional group, the LAS concentration increases in the following order: support < monometallic catalyst < bimetallic catalyst. Modification of Sibunit by carboxyl groups leads to almost complete abolishment of LAS. However, after palladium deposition, their concentration increases by 11 fold. At the same time, application of a bimetallic system leads to an increase in LAS concentration by only 2.3 fold in comparison with the corresponding support.

The analysis of the obtained results indicates the predominance of Brønsted acid and basic sites (BAS and BBS) on the surface of all the studied samples. In the case of unmodified samples, the concentration of all types of sites slightly varies between the support and the corresponding mono- and bimetallic catalysts. However, for the modified supports, the redistribution of acid and basic sites is observed after palladium or bimetallic Pd-Au system deposition. At the same time, the character of the change in acid–base properties depends on the nature of the functional groups (COOH, butyl or NH_2_).

### 3.6. HRTEM Results

Figure 5 shows HRTEM images and EDX maps of the investigated catalysts, as well as histograms of palladium nanoparticles (Pd NPs) and bimetallic Pd-Au nanoparticle (Pd-Au NP) distribution on the surface of these catalysts.

The distribution of Pd NPs on the unmodified Sibunit surface, as well as modified by butyl, carboxyl and amino groups, is approximately the same and ranges from 2 to 9 nm, with the average size of Pd NPs being 4.2 nm for Pd/Cp and Pd/Cp-COOH catalysts, 4.6 nm for Pd/Cp-butyl and 4.4 nm for Pd/Cp-NH_2_. The bimetallic catalysts are characterized by a smaller average nanoparticle size and narrower distribution compared to the corresponding monometallic systems. The smallest average size of NPs was found on the surface of the Pd-Au/Cp-butyl sample (3.2 nm). The average size of Pd-Au NPs in the case of Pd-Au/Cp, Pd-Au/Cp-COOH and Pd-Au/Cp-NH_2_ catalysts was 3.9, 3.6 and 3.4 nm, respectively. A possible explanation for the formation of smaller NPs in the case of bimetallic catalysts may be the shorter metal–metal bond length for Pd-Au (2.50 Å) compared with the bond length for Pd-Pd (2.74 Å), which in turn leads to the formation of NPs with higher tightly packed crystal lattice [42,43]. The EDX maps show clear evidence of the formation of bimetallic Pd-Au NPs. According to the data presented, palladium and gold are localized on the support surface in close proximity to each other.

### 3.7. XPS Results

The electronic states of palladium, gold (in the case of bimetallic catalysts), oxygen and carbon on the surface of the investigated materials were assessed by XPS.

**Figure 6:**
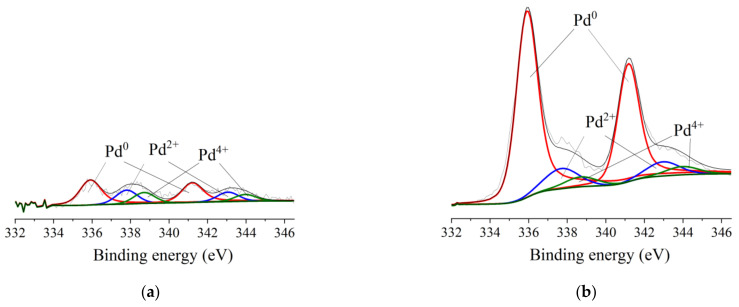

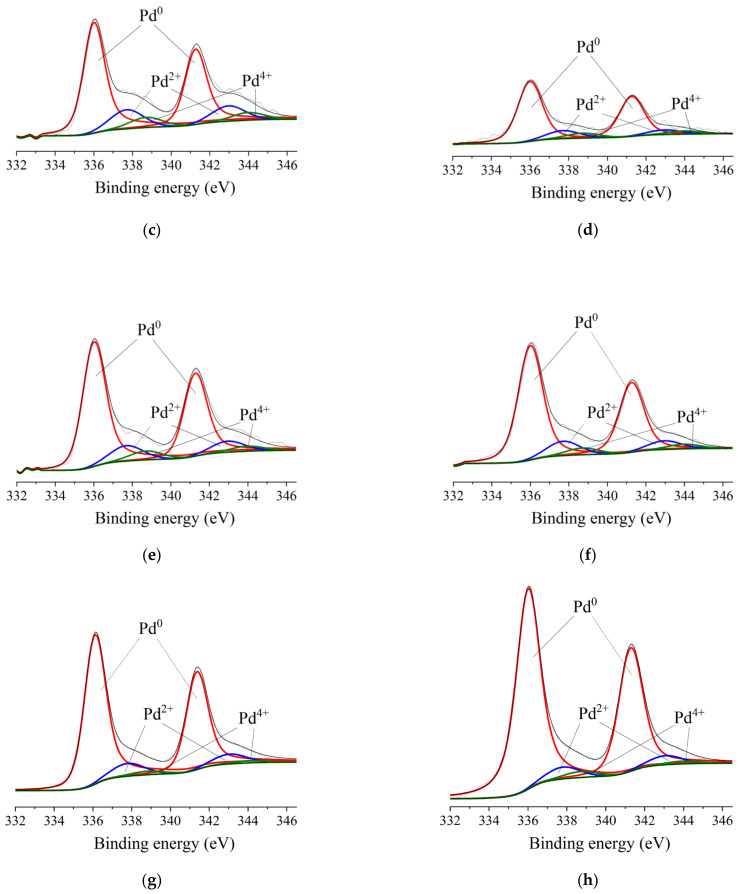
Pd3d XPS spectra: (**a**) Pd/Cp; (**b**) Pd-Au/Cp; (**c**) Pd/Cp-COOH; (**d**) Pd-Au/Cp-COOH; (**e**) Pd/Cp-butyl; (**f**) Pd-Au/Cp-butyl; (**g**) Pd/Cp-NH_2_; (**h**) Pd-Au/Cp-NH_2_.

The Pd3d XPS spectra are shown in Figure 6. Analysis of the spectra demonstrates that palladium is present in the following three states on the surface of all catalysts: Pd^0^, Pd^2+^ and Pd^4+^ with binding energies (Pd3d_5/2_) 335.9—336.1, 337.7—337.8 and 338.7 eV, respectively [44,45,46,47]. It is worth noting that the binding energy values of 335.9 and 336.0 eV, which refers to the Pd^0^ state in the current study, are 0.5 and 0.6 eV higher than the standard BE value characterizing the Pd^0^ state (335.4 eV), which indicates the presence of highly dispersed metal particles on the surface of the studied samples, for which a shift toward higher binding energies up to 1 eV is possible [48,49,50,51].

The contribution of different electronic states of palladium on the surface of the investigated samples, determined by deconvolution of the Pd3d spectrum, is presented in Table 5. The data show that, for most catalysts, the ratio between different states of palladium is approximately the same, except for the Pd/Cp sample, for which 41% of palladium is in the oxidized state (Pd^2+^ and Pd^4+^), while for other catalysts, this value does not exceed 22%, and increases in the following order: Pd/Cp-NH_2_ and Pd-Au/Cp-NH_2_ < Pd-Au/Cp < Pd/Cp-butyl and Pd-Au/Cp-butyl < Pd/Cp-COOH and Pd-Au/Cp-COOH. It should be separately noted that surface palladium concentration for unmodified catalysts (Table 6) is only 0.1–0.2 at.%, whereas, for other samples, this value varies from 0.8 to 2.7 at.% and increases in the following order: COOH < butyl < NH_2_. The low surface concentration of palladium on the surface of Pd/Cp and, accordingly, the low intensity of the signal, are probably due to the non-uniform distribution of Pd NPs on the support surface resulting in their local accumulation, as evidenced by EDX maps (Figure 5).

**Figure 7:**
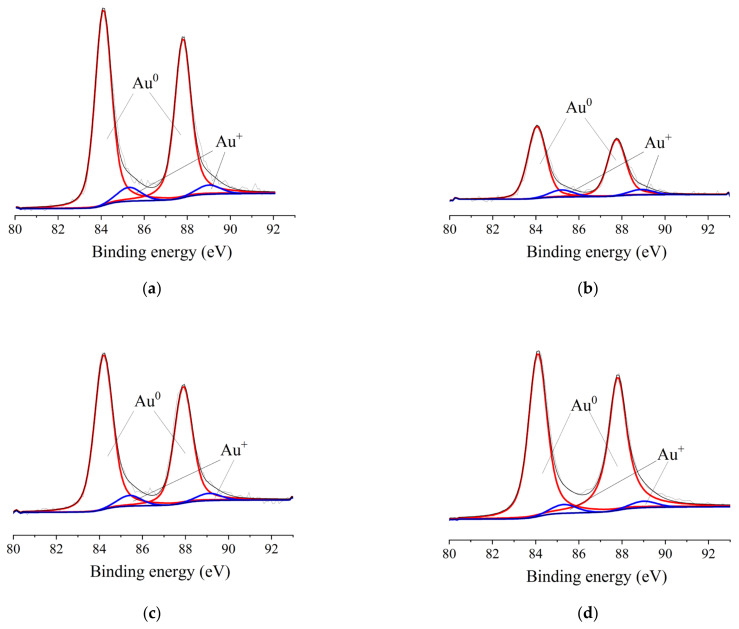
Au4f XPS spectra of (**a**) Pd-Au/Cp, (**b**) Pd-Au/Cp-COOH, (**c**) Pd-Au/Cp-butyl and (**d**) Pd-Au/Cp-NH_2_.

Figure 7 shows the Au4f XPS spectra. According to the presented data, gold is in the following two states on the surface of all bimetallic catalysts: Au^0^ and Au^+^, with bonding energies (Au4f_7/2_) 84.1–84.2 and 85.2–85.4 eV, respectively. The ratio between these states changes insignificantly when the surface of Sibunit is modified by butyl, carboxyl and amino groups (Table 5). However, the surface concentration of gold, for the studied catalysts, varies in the range of 0.2–1.23 at.% (Table 6) and increases as follows: Pd-Au/Cp-COOH < Pd-Au/Cp < Pd-Au/Cp-butyl < Pd-Au/Cp-NH_2_.

For all studied catalysts, the O1s peak deconvolved into four states (Figure S1, Supplementary Materials) related to oxygen atoms within carbonyl groups with BE (O1s) = 531.5–531. 6 eV (C=O); oxygen atoms bound in single bonds to carbon atoms with BE O1s) = 532.5–532.7 eV (C-O); oxygen atoms in hydroxyl groups with BE (O1s) = 533.7–533.9 eV (C-OH) and in carboxyl groups and/or adsorbed water with BE (O1s) = 535.0–535.1 eV (O in H_2_O or COOH) [52,53,54]. The relative contribution of each oxygen state is presented in Table 7. The main contribution is made by oxygen bound in single bonds with carbon, with the content in the catalyst varying from 46% (Pd/Cp-NH_2_) to 65% (Pd/Cp). The fraction of oxygen bound to hydrogen and carbon in the hydroxyl group varies from 16% (Pd/Cp) to 32% (Pd/Cp-COOH and Pd/Cp-NH_2_). For the samples modified by carboxyl and amino groups, the fraction of oxygen as part of the C-OH groups is less for bimetallic catalysts; for unmodified samples, this fraction is smaller for the monometallic sample. In the case of butyl containing mono- and bimetallic catalysts, the fraction of oxygen in the composition of C-OH groups is almost the same. The relative content of oxygen bound by a double bond with carbon (C=O) is the highest for the Pd-Au/Cp-COOH catalyst (19%), and smallest for Pd/Cp-COOH (9%). For all other catalysts, the oxygen content of the C=O group is approximately the same and varies from 12 to 16%. The amount of oxygen in the form of adsorbed water and/or carboxyl groups (O in H_2_O or COOH) does not exceed 9% for all the samples. It is worth noting separately that the highest surface oxygen concentration was found for Pd/Cp-NH_2_ (16.8 at.%) and Pd-Au/Cp-NH_2_ (13.1 at.%) catalysts, followed by Pd/Cp-COOH (7.1 at.%) and Pd-Au/Cp-COOH (5.9 at.%), for Pd/Cp-butyl (4.5 at.%) and Pd-Au/Cp-butyl (4.2 at.%); unmodified mono- and bimetallic catalysts have the lowest oxygen concentration of 3.8 and 2.9 at.%, respectively (Table 6).

The C1s XPS spectra of the studied catalysts are shown in Figure S2, Supplementary Materials. The C1s peaks were deconvolved into five components characterizing the carbon states in C-C (284.8 eV), C-O (285.5–285.6 eV), C=O (286.7–286.9 eV), O-C=O (288.7–288.9 eV) and π-π* (291.0–291.2 eV) [54,55,56,57,58]. Based on the analysis of the contributions of the different carbon states (Table S3), the following conclusions can be drawn: the main contribution is made by C-C, the relative carbon content in this state varies from 64 to 70%; the carbon content of the oxygen-containing functional groups varies from 25–31%, and 3–5% in the π-π* bonds. It is important to note that the C=O value for the NH_2_-modified samples is the highest, compared to the other samples, due to the possible overlapping peaks of the C=O and N-C=O functional groups, which are approximately in the same range of the binding energies [59]. In general, modifying the Sibunit surface or introducing gold along with palladium has little effect on changing the contribution of the different carbon states.

### 3.8. Hydrogenation of Furfural

**Scheme 1:**
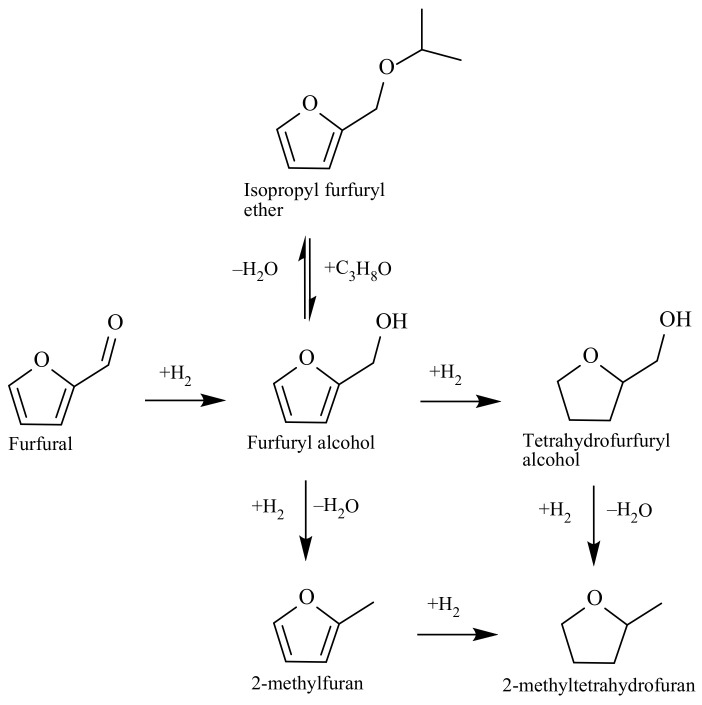
Possible products of the furfural hydrogenation.

**Figure 8:**
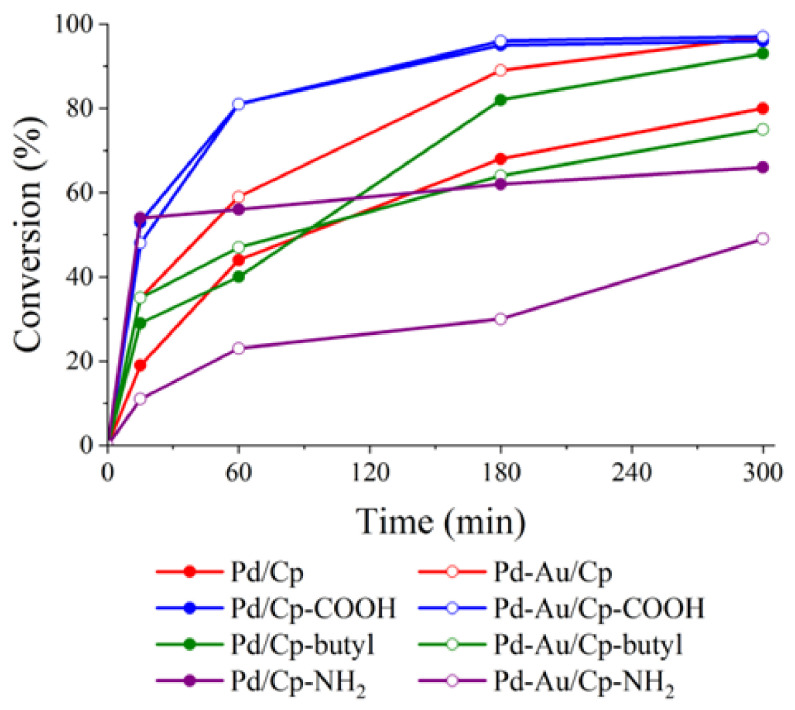
Conversion of furfural in hydrogenation processes over different catalysts. Reaction conditions: 0.3 M furfural (0.2882 g) in 10 mL 2-propanol, amount of catalyst 0.0645 g (Pd/furfural = 1:500 mol/mol; Pd-Au/furfural = 1:580 mol/mol), T = 50 °C, p (H2) = 5 bar, t = 5 h, stirring 1000 rpm.

**Figure 9:**
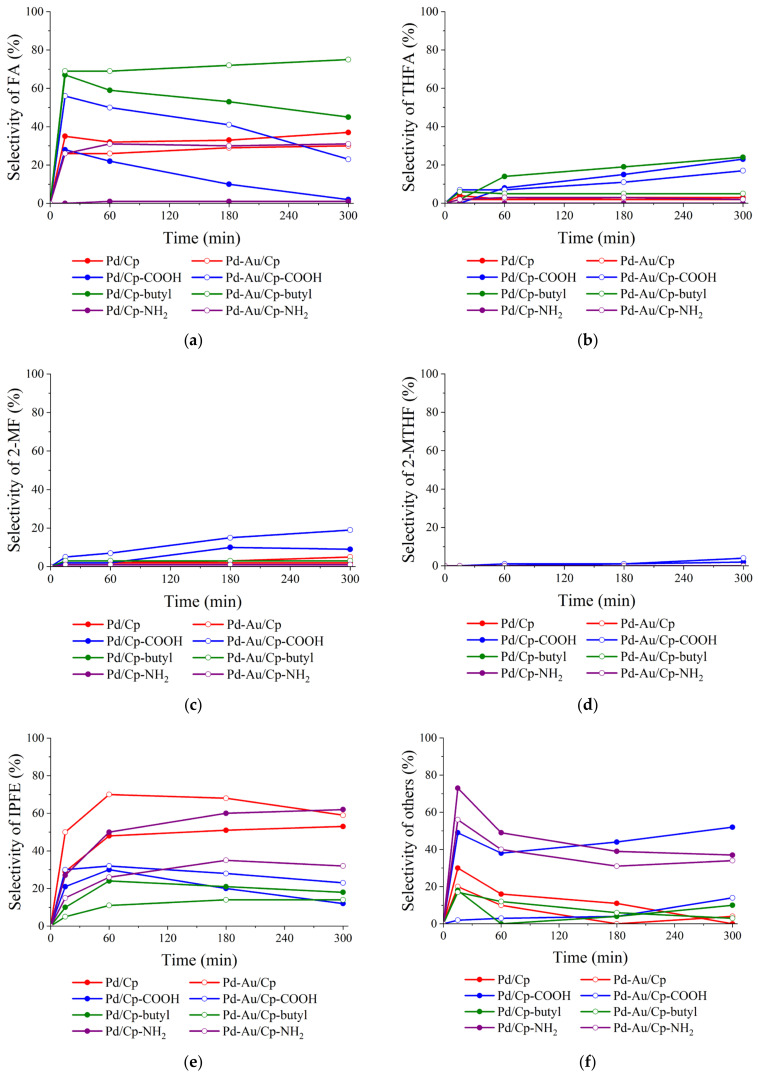
Time evolution of catalytic hydrogenation of furfural: (**a**) furfuryl alcohol; (**b**) tetrahydrofurfuryl alcohol; (**c**) 2-methylfuran; (**d**) 2-methyltetrahydrofuran; (**e**) isopropyl furfuryl ether; (**f**) others. Reaction conditions: 0.3 M furfural (0.2882 g) in 10 mL 2-propanol, amount of catalyst 0.0645 g (Pd/furfural = 1:500 mol/mol; Pd-Au/furfural = 1:580 mol/mol), T = 50 °C, pH_2_ = 5 bar, t = 5 h, stirring 1000 rpm.

The catalytic behavior of the supported mono- and bimetallic unmodified and modified catalysts was evaluated in the reaction of liquid-phase hydrogenation of furfural at 50 °C and 5 bar H_2_ (Table 7, Scheme 1, Figure 8 and Figure 9).

The highest furfural conversion (96–97%) was achieved on Pd/Cp-COOH, Pd-Au/Cp-COOH and Pd-Au/Cp (Table 7, entries 3, 5 and 7, Figure 8). However, despite the high activity (furfural conversion), the selectivity for the desired products (furfuryl alcohol, tetrahydrofurfuryl alcohol, 2-methylfuran, 2-methyltetrahydrofuran) using these catalysts did not exceed 67% (Table 7, entries 3, 5 and 7, Figure 9). It is worth noting that, for Pd/Cp-COOH, more than 40% of the reaction products were unidentified (Table 7, entry 3, Figure 9) and for Pd-Au/Cp, the main hydrogenation product was isopropyl furfuryl ether, formed as a result of the interaction between the reagent (furfuryl) and the solvent (isopropyl alcohol) (Table 7, entry 5, Figure 9). For Pd/Cp, the conversion of furfural was 80% (Table 7, entry 1, Figure 9) and the distribution of reaction products for this sample was similar to Pd-Au/Cp (Table 7, entry 5, Figure 9). In the case of the monometallic sample modified by butyl groups, the conversion of furfural was 93% with a wide distribution of reaction products, among which, furfural and tetrahydrofurfural accounted for 55% and 18%, respectively (Table 7, entry 2, Figure 9). When gold was introduced into this catalyst, along with palladium, the selectivity changed dramatically and furfuryl alcohol was the major product of the reaction (Table 7, entry 6, Figure 9). Overall, by evaluating the reaction product distribution at the same conversion level (70%), this catalyst was the most selective for the desired products compared to the other studied samples. At the same time, it should be noted that this sample had the lowest conversion of furfural (75%) among the studied catalysts. The least active and selective were catalysts on the basis of Pd and Pd-Au NPs supported on Sibunit modified with amino groups (Table 7, Figure 4 and Figure 9). The highest conversion achieved for these samples was 66%. The bimetallic catalyst is more selective for the main products but less active than the corresponding monometallic catalyst.

## 4. Conclusions

The core idea of the present study is to show that the catalytic behavior (activity and selectivity) of furfural hydrogenation catalysts can be influenced not only by changing the reaction parameters [60] or the content of supported metals [61], but also by changing the surface chemistry of the support, if all other conditions are kept equal. By analyzing the above results of the physicochemical and catalytic studies, it can be concluded that the most significant variation in the physicochemical properties of catalysts, after modification of the support, is observed in a change in the average size of metal particles and its distribution, which in turn affects the catalytic behavior of the studied materials. At the same time, it should be taken into account that the nature of the functional groups (butyl, carboxyl or amino group) plays an equally important role alongside the particle size and its distribution. The most striking example, in this case, is a comparison of the catalytic characteristics for Pd-Au/Cp-butyl and Pd-Au/Cp-NH_2_ with close NP size Pd-Au NPs, but very different catalytic performances (furfural conversion and selectivity). It is worth noting separately that no correlation between the structural (Figure 4), textural (Table 3), acid–base (Table 4), electronic (Table 5, Tables S2 and S3; Figure 6 and Figure 7, Figures S1 and S2) and catalytic properties (Table 7, Figure 8 and Figure 9) of the studied materials was found. The structural and electronic properties of the catalysts were the least affected by the modification. The analysis of the kinetic curves revealed the considerable effect of surface functional groups on activity and selectivity, which cannot be explained by the identified minor changes in the physicochemical properties of the studied catalysts. Thus, we hypothesize that the selectivity can be achieved by specific interactions of intermediates with functional groups associated with electrostatic and/or hydrophobic/hydrophilic binding. At the same time, the in-depth evaluation of the mechanism obviously requires a comprehensive study, which is outside the scope of this paper. Nevertheless, the main conclusion of this study is the possibility to finely tune the performance of a catalyst, including the selectivity, by appropriate modification of the carbon support.

## Figures and Tables

**Table 1:** Catalytic hydrogenation of furfural on different palladium catalysts.

Catalyst	Reaction Conditions	Conv., %	Selectivity, %				Ref.
			FA	THFA	2-MF	2-MTHF	
1% Pd/TiO_2_	RT, 3 bar H_2_, 0.1 g of catalyst, 1 g of furfural, octane—solvent, 2 h	20.7	73	14	7	-	[27]
2.5 % Pd— 2.5 % Ru/TiO_2_	RT, 3 bar H_2_, 0.1 g of catalyst, 1 g of furfural, octane—solvent, 2 h	33.8	58	0.4	14	-	[27]
5% Pd/C	150 °C, 2.0 MPa H_2_, 800 rpm, 0.4 g of catalysts, 9.6 g of furfural, 6 g of acetic acid, toluene—solvent, 4 h	41.2	35	-	21.6	-	[28]
5 % Pd/Al_2_(SiO_3_)_3_	150 °C, 2.0 MPa H_2_, 800 rpm,0.4 g of catalysts, 9.6 g of furfural, 6 g of acetic acid, toluene—solvent, 4 h	56.9	52.7	-	-	-	[28]
1% Pd/CNT	180 °C, 2.0 MPa H_2_, 600 rpm, 0.01 g of catalysts, 1 g of furfural, 2-propanol—solvent, 5 h	29.1	14.9	2.5	1.8	-	[29]
1% Pd/Vulcan	180 °C, 2.0 MPa H_2_, 600 rpm,0.01 g of catalysts, 1 g of furfural, 2-propanol—solvent, 5 h	46.7	24.3	7.2	1.8	-	[29]
1% Pd/CMK-5	180 °C, 2.0 MPa H_2_, 600 rpm,0.01 g of catalysts, 1 g of furfural, 2-propanol—solvent, 5 h	100	20.3	31.5	13.4	-	[29]
Pd/C + CuO	170 °C, 3.0 MPa H_2_, 500 rpm, 0.3 g of furfural, 1,4-dioxane—solvent, 3 h	24.1	61.4	5.8	-	-	[30]
0.9 % Pd/ZrO_2_	30 °C, 0.5 MPa H_2_, 0.05 g of catalysts, 0.1 mL of furfural, water—solvent, 3 h	34	21	16	-	-	[31]
0.8 % Pd/SiO_2_	30 °C, 0.5 MPa H_2_, 0.05 g of catalysts, 0.1 mL of furfural, water—solvent, 3 h	63	39	21	-	-	[31]
1 % Pd/MgAlO_x_	150 °C, 3.0 MPa H_2_, 0.5 g of catalysts, 5.0 cm^3^ of furfural, water—solvent, 1 h	62.9	48.6	32.4	-	-	[32]

**Table 2:** Pd and Au contents in the catalysts.

Catalyst	Content of Element, %	
	Pd	Au ^a^
Nominal	0.7–1.0 ^b^	0–0.3 ^c^
Pd/Cp	0.72	0
Pd-Au/Cp	0.73	0.32
Pd/Cp-COOH	0.93	0
Pd-Au/Cp-COOH	0.63	0.36
Pd/Cp-butyl	0.71	0
Pd-Au/Cp-butyl	0.58	0.42
Pd/Cp-NH_2_	0.92	0
Pd-Au/Cp-NH_2_	0.78	0.28

^a^ determined by XEDS; ^b^ content of Pd is 1.0 and 0.7% for mono- and bimetallic catalysts, respectively; ^c^ content of Au is 0 and 0.3% for mono- and bimetallic catalysts, respectively.

**Table 3:** Textural properties of supports and corresponding catalysts.

Entry	Sample	BET Surface Area, m^2^ g^−1^	Surface Area of Mesopores, m^2^ g^−1^	Surface Area of Micropores, m^2^ g^−1^	Mesopore Volume, cm^3^ g^−1^	Micropore Volume, cm^3^ g^−1^	Mesopore Size, nm	Micropore Size, nm
1	Cp	281	310	7	0.48	0.003	6.2	1.9
2	Pd/Cp	314	357	5	0.59	0.002	6.6	2.0
3	Pd-Au/Cp	327	368	5	0.60	0.003	6.5	2.0
4	Cp-COOH	209	264	-	0.45	-	6.8	-
5	Pd/Cp-COOH	228	280	2	0.45	0.001	6.4	1.9
6	Pd-Au/Cp-COOH	252	302	4	0.52	0.002	6.9	1.9
7	Cp-butyl	246	307	4	0.53	0.002	6.9	1.8
8	Pd/Cp-butyl	237	299	3	0.49	0.002	6.5	1.8
9	Pd-Au/Cp-butyl	258	321	3	0.52	0.001	6.5	1.8
10	Cp-NH_2_	235	294	4	0.48	0.002	6.5	1.8
11	Pd/Cp-NH_2_	235	291	3	0.48	0.002	6.0	1.6
12	Pd-Au/Cp-NH_2_	274	326	6	0.48	0.003	5.9	1.8

**Table 4:** The concentration of acid–base centers on the surface of the studied catalysts and corresponding supports (q, μmol/g): 1—Cp; 2—Pd/Cp; 3—Pd-Au/Cp; 4—Cp-COOH; 5—Pd/Cp-COOH; 6—Pd-Au/Cp-COOH; 7—Cp-butyl; 8—Pd/Cp-butyl; 9—Pd-Au/Cp-butyl; 10—Cp-NH_2_; 11—Pd/Cp-NH_2_; 12—Pd-Au/Cp-NH_2_.

pK_a_	SAMPLE											
	1	2	3	4	5	6	7	8	9	10	11	12
**LEWIS BASE**												
**−0.29**	13.7	14.5	13.4	5.9	13.8	13.3	10.9	11,5	10.3	14.3	14.5	14
**BRØNSTED ACID**												
**0.71**	7.0	6.8	6.9	5.1	5.1	5.7	4.9	6.1	7.0	7.2	7.2	7.1
**1.30**	3.5	2.6	3.5	2.0	3.4	3.4	2.0	1.6	2.0	3.1	2.7	3.6
**2.50**	6.7	7.1	7.0	0.0	2.5	4.5	5.6	1.7	2.8	0.1	5.9	5
**3.46**	6.6	6.7	7.1	7.1	5.8	7.3	6.9	6.1	6.4	7.2	7	7
**5.00**	6.8	6.7	6.2	7.3	6.5	6.7	6.5	5.7	5.9	2.4	5.5	5.4
**6.40**	2.2	1.9	0.3	0.1	0.1	0.1	0.1	0.1	0.4	0.5	2	2.1
**Σ**	32.8	31.8	31.0	21.6	23.4	27.7	26.0	21.3	24.5	20.5	30.3	30.2
**BRØNSTED BASE**												
**9.45**	14.9	14.4	14.2	4.2	6.8	9.9	6.1	9.2	10.3	13.2	0.8	0.5
**10.50**	3.2	3.6	3.6	3.1	3.4	3.1	3.3	3.0	2.7	3	2.9	3
**12.00**	8.3	7.2	8.9	6.3	7.8	7.7	5.9	5.6	8.2	9.4	8.4	8.7
**Σ**	26.4	25.2	26.7	13.6	18.0	20.7	15.3	17.8	21.2	25.6	12.1	12.2
**LEWIS ACID**												
**17.20**	9.0	7.8	8.3	0.4	4.3	0.9	6.5	8.4	9.6	8.2	7.7	8.1
**TOTAL**												
**Σ**	81.9	79.3	79.4	41.5	59.5	62.6	58.7	59	65.6	68.6	64.6	64.5

**Table 5:** Effect of support modification on the contribution of different electronic states of Pd and Au on the catalyst surface calculated according to XPS.

Catalyst	Pd^(0, 2+ or 4+)^ Relative Content, %			Au^(0 or 1+)^ Relative Content, %	
	Pd^0^ (335.9–336.1 eV)	Pd^2+^ (337.7–337.8 eV)	Pd^4+^ (338.7 eV)	Au^0^ (84.1–84.2 eV)	Au^+^ (85.2–85.4 eV)
Pd/Cp	59	23	18	-	-
Pd-Au/Cp	84	9	7	93	7
Pd/Cp-COOH	78	14	8	-	-
Pd-Au/Cp-COOH	80	12	8	90	10
Pd/Cp-butyl	83	11	6	-	-
Pd-Au/Cp-butyl	83	12	5	93	7
Pd/Cp-NH_2_	89	8	3	-	-
Pd-Au/Cp-NH_2_	91	7	2	92	8

**Table 6:** Surface concentration of elements on the catalyst surface (at.%) determined by XPS.

Catalyst	Element				
	C1s	O1s	N1s	Pd3d	Au4f
Pd/Cp	95.9	3.8	0.07	0.2	-
Pd-Au/Cp	95.3	2.9	0.06	0.1	0.3
Pd/Cp-COOH	91.8	7.1	0.20	1.0	-
Pd-Au/Cp-COOH	93.0	5.9	n.d.	0.8	0.2
Pd/Cp-butyl	94.1	4.5	0.02	1.3	-
Pd-Au/Cp-butyl	94.2	4.2	0.17	1.1	0.4
Pd/Cp-NH_2_	77.1	16.8	1.69	2.7	-
Pd-Au/Cp-NH_2_	81.5	13.1	1.62	2.5	1.23

**Table 7:** Catalytic results of the hydrogenation of furfural.

Entry	Catalyst	d_m_, nm	Conversion for 5 h, %	Selectivity at 70% of Conversion, %					
				FA	THFA	2-MF	2-MTHF	IPFE	Others
1	Pd/Cp	4.2	80	33	3	2	0	51	11
2	Pd/Cp-butyl	4.6	93	55	18	3	0	22	2
3	Pd/Cp-COOH	4.2	96	27	4	2	0	27	41
4	Pd/Cp-NH_2_	4.4	66	1	0	0	0	62	37
5	Pd-Au/Cp	3.9	97	26	2	2	0	69	1
6	Pd-Au/Cp-butyl	3.2	75	74	5	3	0	14	4
7	Pd-Au/Cp-COOH	3.6	97	54	7	6	0	32	1
8	Pd-Au/Cp-NH_2_	3.4	49	31	3	1	0	16	49

FA—furfuryl alcohol; THFA—tetrahydrofurfuryl alcohol; 2-MF—2-methylfuran; 2-MTHF—2 methyltetrahydrofuran; IPFE—isopropyl furfuryl ether; others—furan, tetrahydrofuran, 1,2—pentanediol, etc. Reaction conditions: 0.3 M furfural (0.2882 g) in 10 mL 2-propanol, amount of catalyst 0.0645 g (Pd/furfural = 1:500 mol/mol; Pd-Au/furfural = 1:580 mol/mol), T = 50 °C, pH2 = 5 bar, t = 5 h, stirring 1000 rpm.

## Data Availability

Data available upon request.

## Supplementary Materials

The following supporting information can be downloaded at: https://www.mdpi.com/article/10.3390/ma15134695/s1, Figure S1: O1s XPS spectra of (**a**) Pd/Cp; (**b**) Pd-Au/Cp; (**c**) Pd/Cp-COOH; (**d**) Pd-Au/Cp-COOH; (**e**) Pd/Cp-butyl; (**f**) Pd-Au/Cp-butyl; (**g**) Pd/Cp-NH_2_ and (**h**) Pd-Au/Cp-NH_2_; Figure S2: C1s XPS spectra of (**a**) Pd/Cp; (**b**) Pd-Au/Cp; (**c**) Pd/Cp-COOH; (**d**) Pd-Au/Cp-COOH; (**e**) Pd/Cp-butyl; (**f**) Pd-Au/Cp-butyl; (**g**) Pd/Cp-NH_2_ and (**h**) Pd-Au/Cp-NH_2_; Figure S3: O1s XPS spectra of (**a**) Cp; (**b**) Cp-COOH; (**c**) Cp-butyl and (**d**) Cp-NH_2_; Figure S4: C1s XPS spectra of (**a**) Cp; (**b**) Cp-COOH; (**c**) Cp-butyl and (**d**) Cp-NH_2_; Table S1: Effect of support modification on contribution of different electronic states of oxygen on the support surface calculated according to XPS; Table S2: Effect of support modification on contribution of different electronic states of carbon on the support surface calculated according to XPS; Table S3: Surface concentration of elements on the support surface (at.%) determined by XPSat; Table S4: Effect of support modification on contribution of different electronic states of oxygen on the support surface calculated according to XPS; Table S5: Effect of support modification on contribution of different electronic states of carbon on the support surface calculated according to XPS; Table S6: Surface concentration of elements on the support surface (at.%) determined by XPS.
