# Supplementary material for: The Effect of Sibunit Carbon Surface Modification with Diazonium Tosylate Salts of Pd and Pd-Au Catalysts on Furfural Hydrogenation
Source: Materials (Basel). 2022 Jul 4;15(13):4695. doi: 10.3390/ma15134695 (PMC9267796; doi:10.3390/ma15134695)
Supplement: Supplementary file 1 [file materials-15-04695-s001.zip › materials-1758460-supplementary.pdf]

materials

Supporting information

The effect of Sibunit carbon surface modification with diazonium tosylate salts of Pd and PdAu catalysts on furfural hydrogenation

Dmitrii German [1], Ekaterina Kolobova [1], Ekaterina Pakrieva [1], Sonia A.C. Carabineiro [2,3], Elizaveta Sviridova [1], Sergey Perevezentsev [4], Shahram Alijani [5], Alberto Villa [5], Laura Prati [5], Pavel Postnikov [1], Nina Bogdanchikova [6] and Alexey Pestryakov [1,7*]

1 Research School of Chemistry & Applied Biomedical Sciences, National Research Tomsk Polytechnic University, Lenin Av. 30, 634050 Tomsk, Russia; germandmitry93@gmail.com (D.G.); epakrieva@mail.ru (E.P.); ekaterina_kolobova@mail.ru (E.K.); evs31@tpu.ru (E.S.); postnikov@tpu.ru (P.P.)
2 Centro de Química Estrutural, Institute of Molecular Sciences, Departamento de Engenharia Química, Instituto Superior Técnico, Universidade de Lisboa, Av. Rovisco Pais, 1049-001 Lisboa, Portugal
3 LAQV-REQUIMTE, Department of Chemistry, NOVA School of Science and Technology, Universidade NOVA de Lisboa, 2829-516 Caparica, Portugal; sonia.carabineiro@fct.unl.pt (S.A.C.C.)
4 Institute of Petroleum Chemistry, Russian Academy of Science, Akademichesky Av. 4, 634021 Tomsk, Russia; sap311@yandex.ru (S.P.)
5 Dipartimento di Chimica, Università degli Studi di Milano, via Camillo Golgi 19, 20133 Milano, Italy; sli@dinex.fi (S.A.); Alberto.Villa@unimi.it (A.V.); laura.prati@unimi.it (L.P.)
6 Centro de Nanociencias y Nanotecnología, Universidad Nacional Autónoma de México, 22800 Ensenada, México; nina@cnyn.unam.mx (N.B.)
7 Laboratory of Catalytic and Biomedical Technologies, Sevastopol State University, 299053 Sevastopol, Russia
* Correspondence: pestryakov2005@yandex.ru

Citation: German, D.; Kolobova, E.; Pakrieva, E.; Carabineiro S.A.C.; Sviridova, E.; Perevezentsev, S.; Alijani, S.; Villa, A.; Prati, L.; Postnikov, P.; Bogdanchikova N.; Pestryakov, A. The effect of Sibunit carbon surface modification with diazonium tosylate salts of Pd and PdAu catalysts on furfural hydrogenation. *Materials* **2022**, *15*, 4695. https://doi.org/10.3390/ma15134695

Received: 21 May 2022e
Accepted: 28 June 2022
Published: 4 July 2022

Publisher's Note: MDPI stays neutral with regard to jurisdictional claims in published maps and institutional affiliations.

Copyright: © 2022 by the authors. Licensee MDPI, Basel, Switzerland. This article is an open access article distributed under the terms and conditions of the Creative Commons Attribution (CC BY) license (https://creativecommons.org/licenses/by/4.0/).

This file contains

Four figures:

Figure S1. O1s XPS spectra of (**a**) Pd/Cp; (**b**) Pd-Au/Cp; (**c**) Pd/Cp-COOH; (**d**) Pd-Au/Cp-COOH; (**e**) Pd/Cp-butyl; (**f**) Pd-Au/Cp-butyl; (**g**) Pd/Cp-NH_2 and (**h**) Pd-Au/Cp-NH_2.
Figure S2. C1s XPS spectra of (**a**) Pd/Cp; (**b**) Pd-Au/Cp; (**c**) Pd/Cp-COOH; (**d**) Pd-Au/Cp-COOH; (**e**) Pd/Cp-butyl; (**f**) Pd-Au/Cp-butyl; (**g**) Pd/Cp-NH_2 and (**h**) Pd-Au/Cp-NH_2.
Figure S3. O1s XPS spectra of (**a**) Cp; (**b**) Cp-COOH; (**c**) Cp-butyl and (**d**) Cp-NH_2.
Figure S4. C1s XPS spectra of (**a**) Cp; (**b**) Cp-COOH; (**c**) Cp-butyl and (**d**) Cp-NH_2.

Six tables:

Table S1. Structures and pKa of the used indicators.
Table S2. Effect of support modification on contribution of different electronic states of oxygen on the catalysts surface calculated according to XPS
Table S3. Effect of support modification on contribution of different electronic states of carbon on the catalyst surface calculated according to XPS.
Table S4. Effect of support modification on contribution of different electronic states of oxygen on the support surface calculated according to XPS.
Table S5. Effect of support modification on contribution of different electronic states of carbon on the support surface calculated according to XPS.

Table S6. Surface concentration of elements on the support surface determined by XPS.

Materials and Methods

Fourier transform infrared (FTIR) spectra were recorded using a ThermoScientific FTIR instrument (Nicolet 8700) in the range between 650 and 4000 cm^{-1} at a spectral resolution of 6 cm^{-1}. 1 mg of dried modified Sibunit was mixed with 200 mg of KBr powder in an agar mortar. The mixture was pressed into a pellet under 7 tons of load for 2–4 min, and the spectrum was recorded immediately. A total of 64 accumulative scans were collected. The signal from a pure KBr pellet was subtracted as a background.

Palladium and gold content were measured by inductively coupled plasma atomic emission spectroscopy (ICP-AES) using iCAP 6300 Duo (Thermo Fisher Scientific, Waltham, MA, USA) and energy dispersive spectroscopy (XEDS) using JEOL JEM-2100F (JEOL Ltd., Tokyo, Japan) equipped with an Oxford INCA X-sight system detector (Oxford Instruments, Abingdon, Oxfordshire, UK).

XRD patterns were recorded by Bruker D8 X-ray diffractometer (Bruker Corporation, Billerica, MA, USA) using CuKα as X-ray source (λ = 0.15406 nm) with a scanning range of 2θ = 10 - 90°.

The textural properties were measured using an ASAP 2060 (Micromeritics Instrument Corporation, Norcross, GA, USA) apparatus. Catalysts were preliminarily degassed in vacuum at 300 °C for 5 h. The surface area and pore size distribution were calculated using the Brunauer-Emmett-Teller (BET) equation applied for the adsorption isotherm at the relative pressures between 0.005 to 0.25, Barrett-Joyner-Halenda (BJH) (desorption branch, mesopores) and Horvath-Kawazoe (micropores, cylinder pore geometry) methods.

The acid-base properties of the supports and the corresponding catalysts were studied by the Hammett indicator method using 11 indicators with pKa values in the range from -0.29 to 17.2 (Table S1).

The sizes of Pd or Pd-Au particles were estimated by high resolution transmission electron microscopy (HRTEM) using a JEOL JEM-2100F (JEOL Ltd., Tokyo, Japan) with a sample preparation system. The samples were dispersing into a fine powder and sonicated in hexane at room temperature. Derived suspension was applied on a carbon-coated Cu grid. For each sample, at least 500 particles were counted.

Surface composition and the chemical state of each element were determined by X-ray photoelectron spectroscopy (XPS), performed on a VG Scientific ESCALAB 200A (Thermo Fisher Scientific, Waltham, MA, USA) spectrometer using Al Kα radiation (1486.6 eV) in CEMUP. The charge effect was corrected using the C1s peak (binding energy of 284.8 eV). The CASA XPS software (http://www.casaxps.com/) was used for data analysis.

Table S1. Structures and pKa of the used indicators.

№	Indicator	pKa	Structure
1	2-Nitroaniline	-0.29	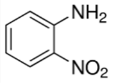
2	2,4,6-Trinitrophenol	0.71	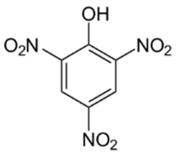

3	Brilliant green	1.30	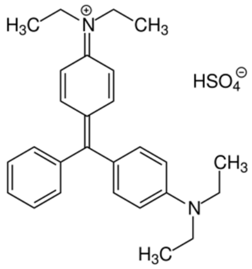
4	3-Nitroaniline	2.50	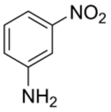
5	Methyl orange	3.46	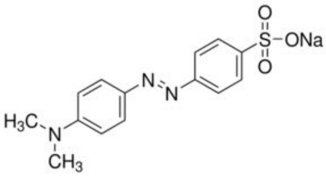
6	Methyl Red	5.00	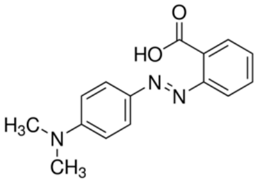
7	Bromocresol purple	6.40	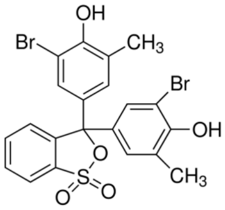
8	Catechol	9.45	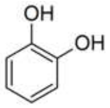
9	Nile blue	10.50	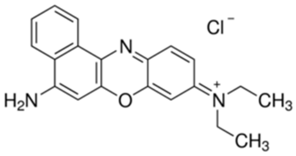
10	Tropaeolin O	12.00	
11	2,4-Dinitrotoluene	17.20	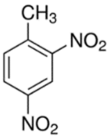

Results

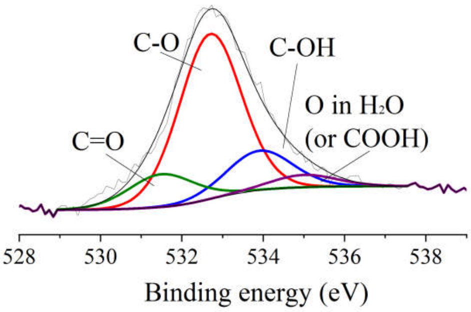

(**a**)

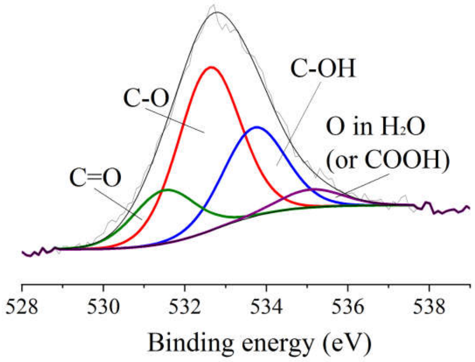

(**b**)

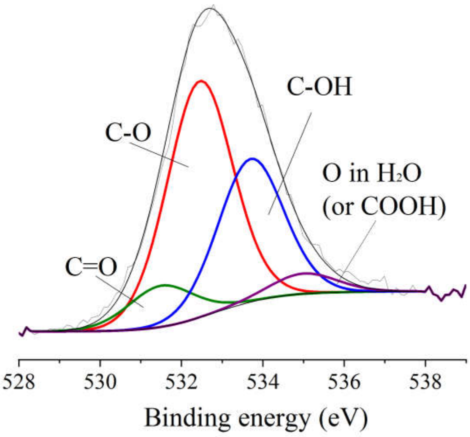

(**c**)

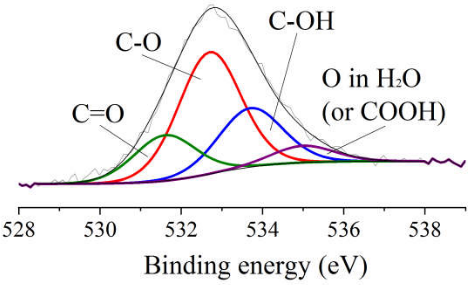

(**d**)

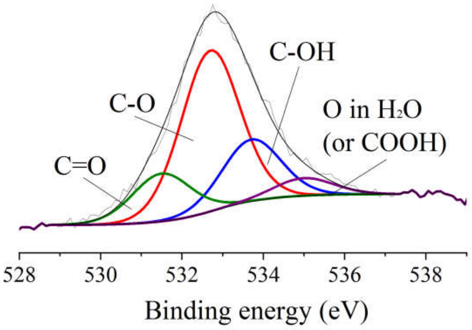

(**e**)

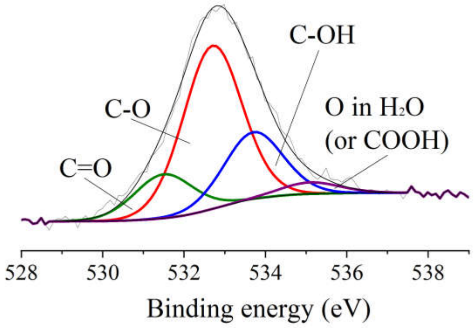

(**f**)

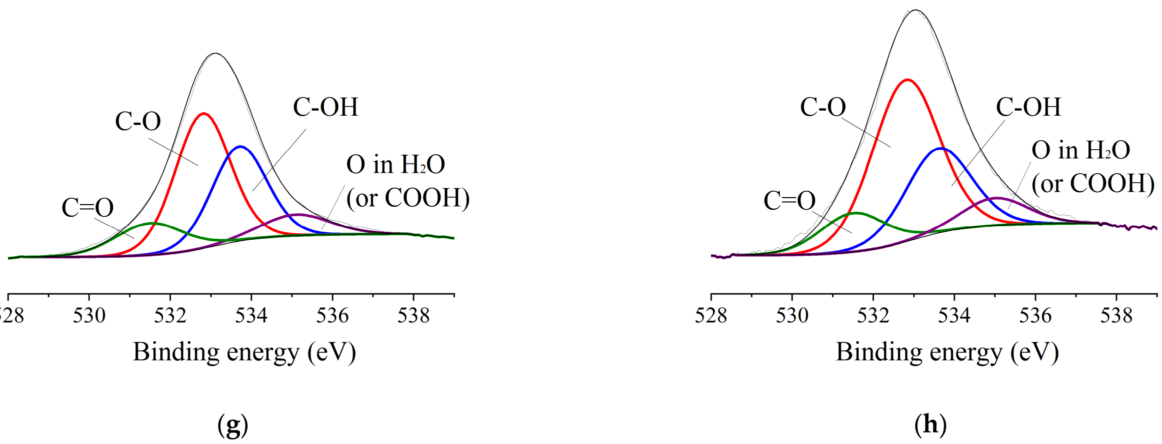

Figure S1. O1s XPS spectra of (**a**) Pd/Cp; (**b**) Pd-Au/Cp; (**c**) Pd/Cp-COOH; (**d**) Pd-Au/Cp-COOH; (**e**) Pd/Cp-butyl; (**f**) Pd-Au/Cp-butyl; (**g**) Pd/Cp-NH_2 and (**h**) Pd-Au/Cp-NH_2.

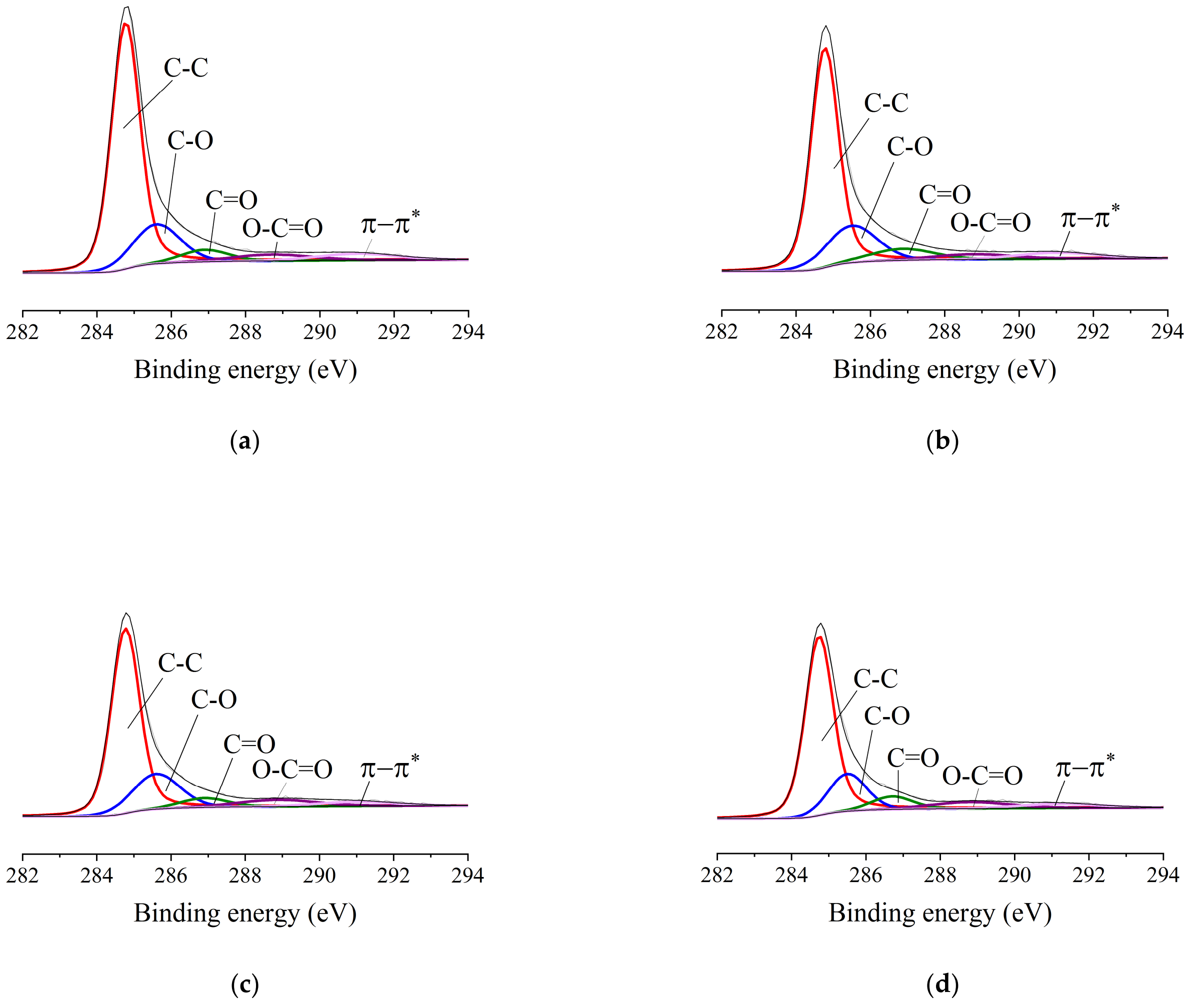

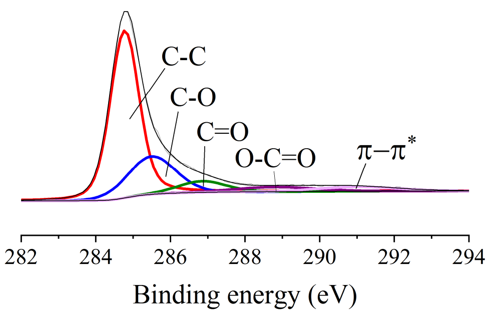

(**e**)

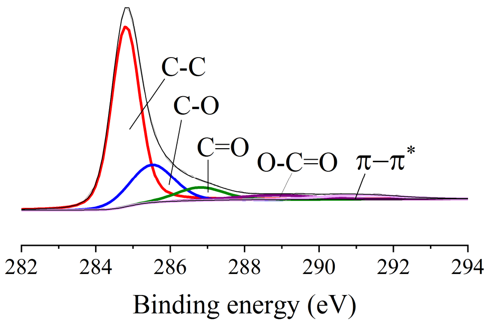

(**f**)

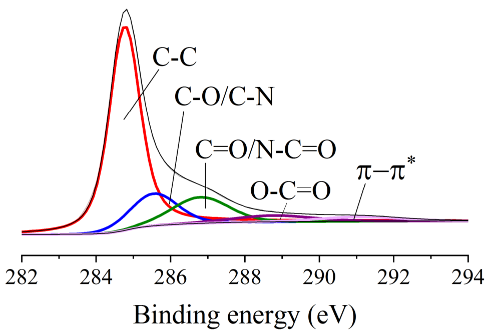

(**g**)

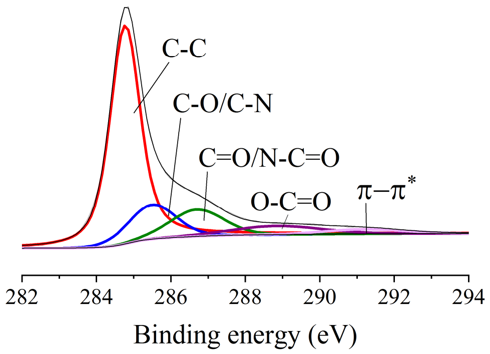

(**h**)

Figure S2. C1s XPS spectra of (**a**) Pd/Cp; (**b**) Pd-Au/Cp; (**c**) Pd/Cp-COOH; (**d**) Pd-Au/Cp-COOH; (**e**) Pd/Cp-butyl; (**f**) Pd-Au/Cp-butyl; (**g**) Pd/Cp-NH_2 and (**h**) Pd-Au/Cp-NH_2.

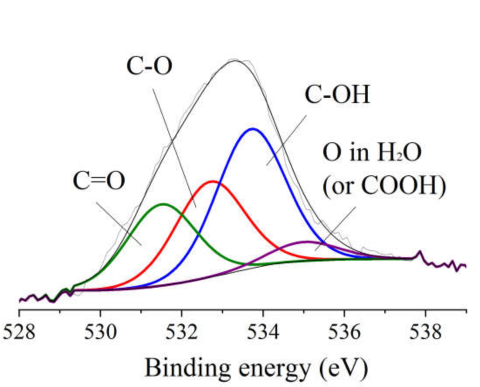

(**a**)

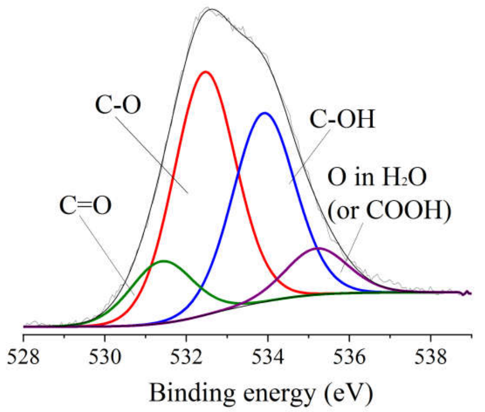

(**b**)

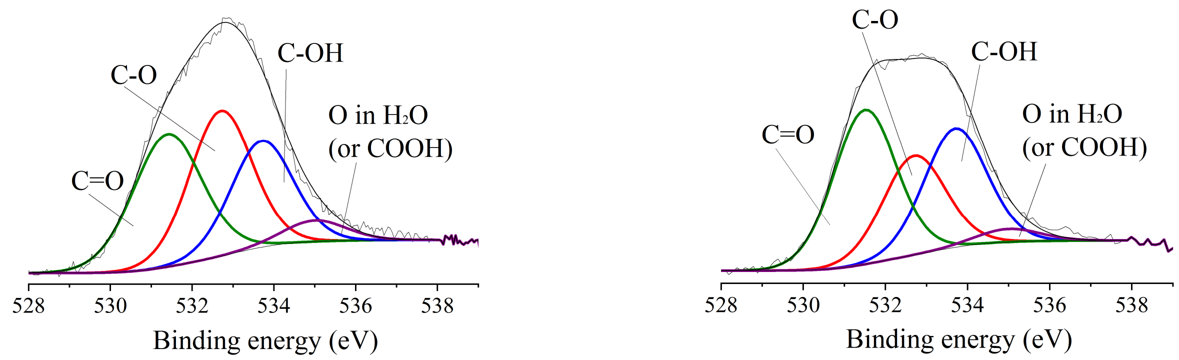

(**c**) (**d**)

Figure S3. O1s XPS spectra of (**a**) Cp; (**b**) Cp-COOH; (**c**) Cp-butyl and (**d**) Cp-NH_2.

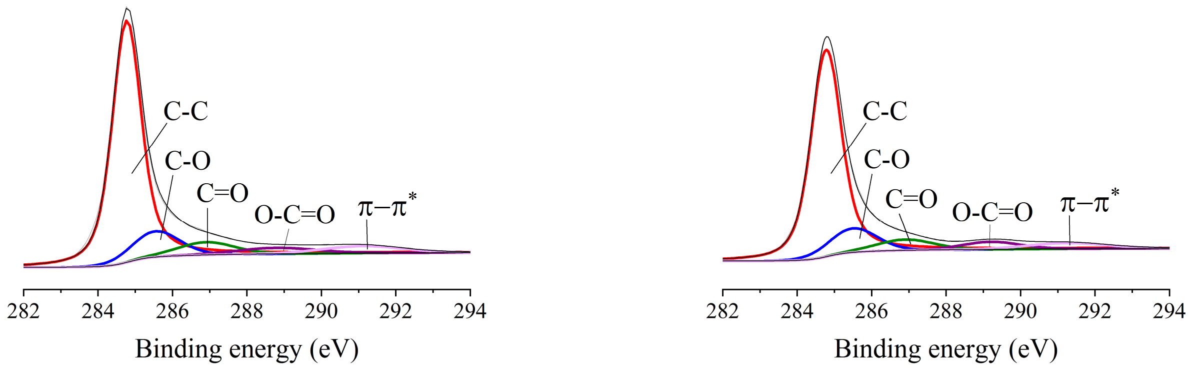

(**a**) (**b**)

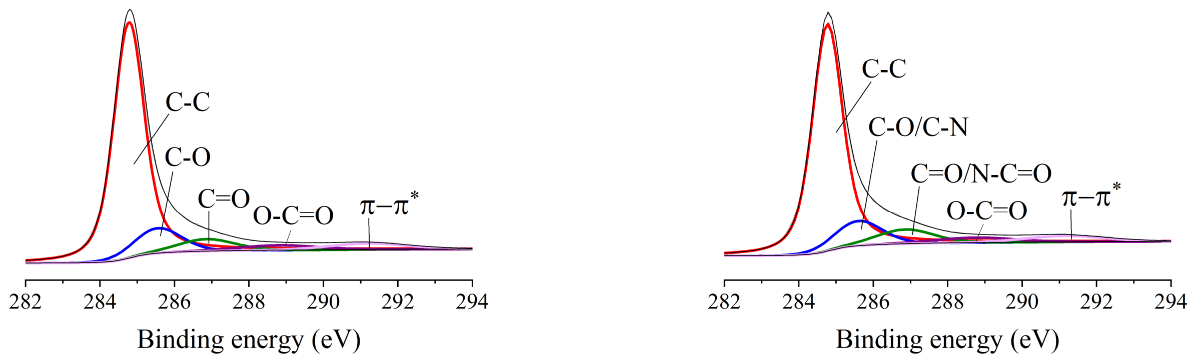

(**c**) (**d**)

Figure S4. C1s XPS spectra of (**a**) Cp; (**b**) Cp-COOH; (**c**) Cp-butyl and (**d**) Cp-NH_2.

Table S2. Effect of support modification on contribution of different electronic states of oxygen on the catalysts surface calculated according to XPS

Catalyst	Oxygen relative content, %			
	C=O (531.5 – 531.6 eV)	C-O (532.5 – 532.7 eV)	C-OH (533.7 – 533.9 eV)	O in H_2O or COOH (535.0 – 535.1 eV)
Pd/Cp	14	65	16	5
Pd-Au/Cp	16	50	28	6
Pd/Cp-COOH	9	54	32	5
Pd-Au/Cp-COOH	19	50	25	6
Pd/Cp-butyl	16	56	22	6
Pd-Au/Cp-butyl	15	57	24	4
Pd/Cp-NH_2	13	46	32	9
Pd-Au/Cp-NH_2	12	52	27	9

Table S3. Effect of support modification on contribution of different electronic states of carbon on the catalyst surface calculated according to XPS.

Catalyst	Carbon relative content, %				
	C-C (284.8 eV)	C-O/C-N (285.5 – 285.6 eV)	C=O/N-C=O (286.7 – 286.9 eV)	O-C=O (288.7 – 288.8 eV)	π-π^* (291.0 – 291.2)
Pd/Cp	70	16	5	4	5
PdAu/Cp	66	18	7	4	5
Pd/Cp-COOH	67	19	5	6	3
PdAu/Cp-COOH	68	17	6	6	3
Pd/Cp-butyl	64	21	6	4	5
PdAu/Cp-butyl	66	19	7	4	4
Pd/Cp-NH_2	67	13	13	4	3
Pd-Au/Cp-NH_2	66	13	12	6	3

Table S4. Effect of support modification on contribution of different electronic states of oxygen on the support surface calculated according to XPS.

Support	Oxygen relative content, %			
	C=O (531.5 – 531.6 eV)	C-O (532.5 – 532.7 eV)	C-OH (533.7 – 533.9 eV)	O in H_2O or COOH (535.0 – 535.1 eV)
Cp	24	28	42	6
Cp-COOH	12	45	35	8
Cp-butyl	35	35	25	5
Cp-NH_2	40	26	31	3

Table S5. Effect of support modification on contribution of different electronic states of carbon on the support surface calculated according to XPS.

**Support**	**Carbon relative content, %**				
	**C-C (284.8 eV)**	**C-O/C-N (285.5 – 285.6 eV)**	**C=O/N-C=O (286.7 – 286.9 eV)**	**O-C=O (288.7 – 288.8 eV)**	**π-π^* (291.0 – 291.2)**
Cp	75	10	6	4	5
Cp-COOH	75	11	6	4	4
Cp-butyl	77	10	6	3	4
Cp-NH_2	75	10	7	3	5

Table S6. Surface concentration of elements on the support surface (at.%) determined by XPS.

**Element**	**Cp**	**Cp-butyl**	**Cp-COOH**	**Cp-NH_2**
C 1s	96.9	96.2	92.2	92.4
O 1s	3.1	3.6	7.6	4.7
S 2p	-	0.2	0.2	0.4
N 1s	-	-		2.5
